# Lumbar Spine Motion Analysis: A Systematic Review of Segmental and Global Range of Motion

**DOI:** 10.7759/cureus.114125

**Published:** 2026-08-07

**Authors:** Qing Hang Tan, Amirzeb Aurangzeb, Hasjmy Mohamad, Dalun Leong, Dinesh Kumar, Michael E Janssen, Zhihong Chew

**Affiliations:** 1 Department of Orthopaedic Surgery, Changi General Hospital, Singapore, SGP; 2 Department of Orthopaedic Surgery, Center for Spine and Orthopedics, P.C., Denver, USA

**Keywords:** lumbar spine, motion analysis, range of motion, segmental and global, systematic review

## Abstract

Accurate assessment of lumbar spine range of motion (ROM) is essential for evaluating spinal biomechanics, monitoring disease progression, and assessing outcomes following motion-preserving interventions. Numerous techniques have been developed to quantify lumbar motion, each differing in measurement principles, accuracy, and clinical applicability. This review synthesizes current methods for assessing segmental and global lumbar spine ROM. A systematic literature search was conducted in PubMed using the terms "lumbar spine" AND "motion analysis" OR "motion evaluation" OR "motion assessment." Peer-reviewed English-language studies published between 2000 and 2025 were screened according to predefined eligibility criteria. Eligible studies were categorized based on assessment methodology and clinical application. Twelve studies met the inclusion criteria and evaluated a range of lumbar spine motion assessment techniques, including dynamic fluoroscopic systems, advanced magnetic resonance imaging (MRI), functional X-ray analysis, motion capture systems, and wearable inertial measurement units (IMU). Dynamic imaging and computer-assisted analysis generally demonstrated improved measurement precision compared with conventional radiographic assessment, while MRI-based and wearable technologies offered radiation-free or ambulatory alternatives. Each modality demonstrated distinct advantages and limitations with respect to measurement accuracy, practicality, and clinical application. A wide range of techniques is available for lumbar spine motion analysis, each offering unique strengths and limitations. Although conventional radiographs remain widely used in clinical practice, emerging technologies provide enhanced dynamic assessment and greater measurement precision. Continued standardization of imaging protocols and further validation of these technologies are needed to facilitate broader clinical implementation.

## Introduction and background

Lower back pain is a ubiquitous global health issue. Lifetime prevalence rates have been reported to be 49% to up to 80% [1]. Degenerative lumbar disc disease underpins a large fraction of this group [2,3]. The primary dichotomy of surgical options is fusion and arthroplasty procedures. While spinal fusion has long been the mainstay of surgical treatment, eliminating motion at the affected segment comes at the expense of altered biomechanics and adjacent-level degeneration, referring to degeneration occurring in spinal segments next to the treated level in up to 27.8% of patients [4]. In response, motion-preserving arthroplasty procedures such as lumbar total disc replacement (LDR) emerged as an alternative. Motion-preserving alternatives have been shown to have significantly lower odds of incidence of adjacent segment disease and reoperations due to adjacent segment disease [5].

Central to the evaluation of spinal biomechanics and lumbar disorders is the accurate assessment of vertebral motion. Quantification of lumbar spine range of motion is important for the diagnosis of spinal instability, evaluation of degenerative conditions, monitoring of treatment outcomes, and assessment of spinal implants, including motion-preserving technologies. Accurate lumbar spine motion analysis has broad clinical and research applications, including clinical diagnosis and functional assessment, treatment evaluation and patient selection, and biomechanical research and implant evaluation.

Clinical diagnosis and functional assessment

Global lumbar motion reflects overall spinal mobility and functional status, whereas segmental motion analysis assists in detecting abnormal intervertebral kinematics associated with spinal instability and degenerative disorders.

Treatment evaluation and patient selection

Motion analysis provides objective assessment of functional recovery following conservative treatment or surgery and may assist in selecting appropriate candidates for motion-preserving or fusion procedures.

Biomechanical research and implant evaluation

Quantification of segmental motion, intervertebral translation, and center of rotation facilitates evaluation of spinal biomechanics and the performance of motion-preserving implants while also improving understanding of normal and pathological lumbar kinematics.

Lumbar spine motion can be assessed at both the global and segmental levels. Global motion reflects overall lumbar mobility and functional status, whereas segmental motion evaluates intervertebral kinematics, providing important information on spinal stability, degenerative changes, and implant performance. As motion analysis techniques differ in their ability to assess these parameters, understanding their respective applications is essential for selecting the appropriate method in clinical practice and research [6].

Practically, standard flexion-extension radiographs remain ubiquitous in clinical practice. This is mainly due to cost and ease of obtaining these images. However, these standard radiographs suffer from inter- and interobserver errors [7], parallax distortion, an imaging error caused by changes in viewing angle that may affect the accuracy of spinal alignment and motion measurements [8], and inability to capture functional loading. Many protocols have been suggested in the literature to standardize methods of obtaining these radiographs.

In the absence of a universally accepted "gold standard" for measuring lumbar spinal segmental motion metrics (range of motion, translation, and rotation), new modalities of treatment will be difficult to assess.

Over the years, development in advanced methods in the pursuit of lumbar spine motion analysis has yielded multiple modalities of assessment. Multiple methods have been proposed and evaluated in the literature. These methods include fluoroscopic techniques with varying methods of tracking and processing the images, such as the Vertebral Motion Analysis^TM^ (VMA^TM^) [9], and utilizing magnetic resonance imaging (MRI) for dynamic imaging [10,11]. Software to assist with the measurement of functional X-ray images has also emerged, such as Functional X-ray Analysis^TM^ (FXA^TM^) software (ACES GmbH, Esslingen, Germany) [12].

Other emerging solutions include wearable inertial sensors with inertial measurement units (IMU) [13], marker-based motion capture systems, which have been developed to provide non-invasive, real-time assessment of lumbar spine motion. Continued technological developments may further improve the accuracy and clinical applicability of these techniques.

In this systematic review article, we intend to synthesize current concepts in lumbar spinal motion analysis. We will explore and summarize recent reports regarding the options available currently for the assessment of lumbar spinal motion.

## Review

Materials and methods

Search Strategy

A systematic literature search was conducted in PubMed following the Preferred Reporting Items for Systematic Reviews and Meta-Analyses (PRISMA) 2020 guidelines [14]. The following search strategy was used: ("lumbar spine"[MeSH Terms] OR "lumbar spine"[Title/Abstract]) AND ("motion analysis"[Title/Abstract] OR "motion evaluation"[Title/Abstract] OR "motion assessment"[Title/Abstract]). The search was limited to English-language, peer-reviewed studies published between January 1, 2000, and March 31, 2025, involving human or animal models. Reference lists of included studies were also screened to identify additional relevant publications.

Eligibility Criteria

Eligible studies were original investigations that assessed, described, validated, or compared methods for measuring segmental or global lumbar spine motion. Studies were excluded if they were not related to lumbar spine motion assessment, were not original research, were conference abstracts, editorials, letters, or narrative reviews, or lacked sufficient methodological detail or outcome data for extraction.

Data Extraction

**Figure 1:**
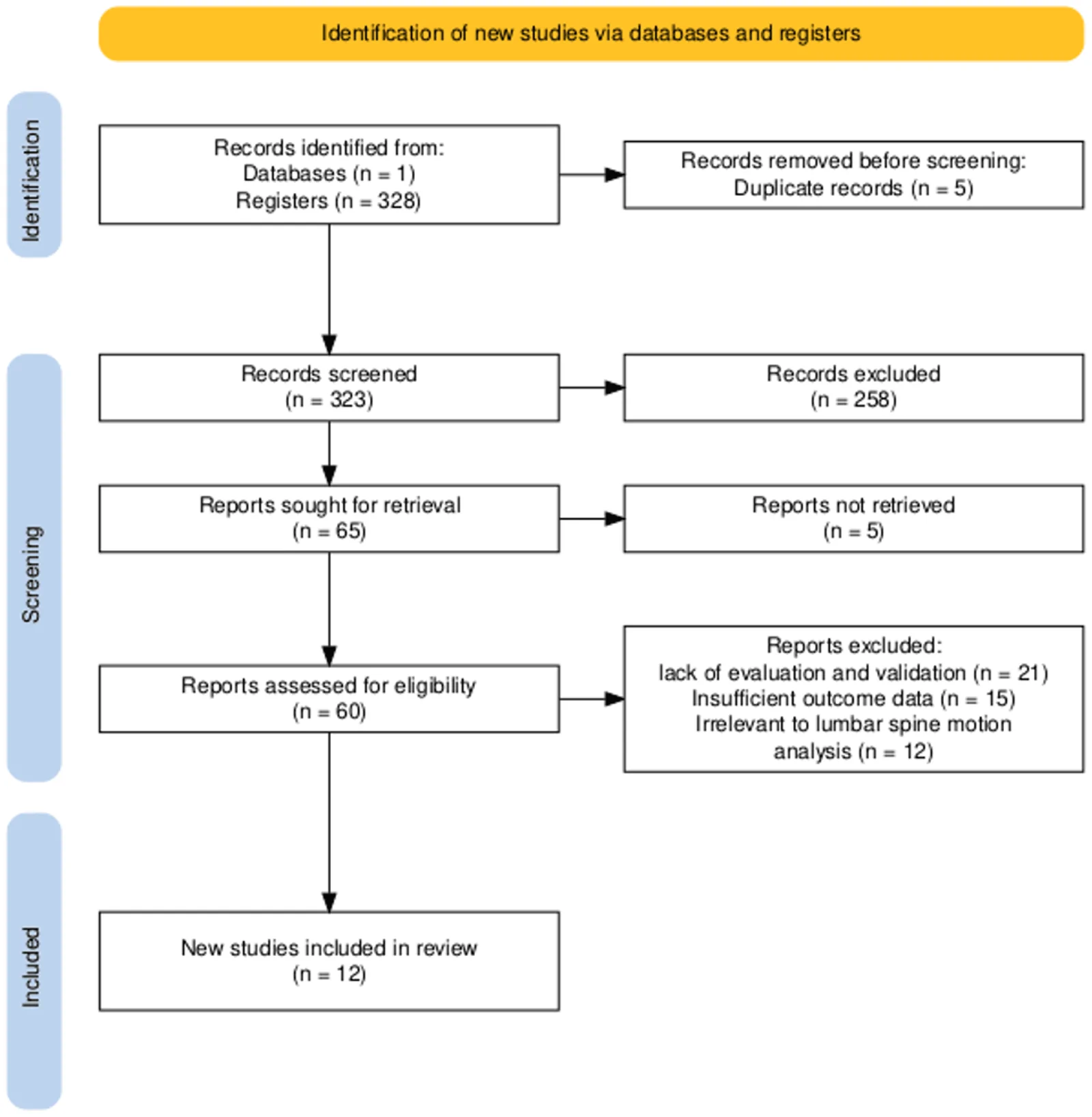
Flow diagram of literature review

Two reviewers independently extracted data using a standardized data extraction form. The extracted information included study design, participant characteristics, sample size, motion analysis modality, measurement methodology, comparator methods, and principal findings. Any discrepancies were resolved through consensus discussion. The heterogeneity of the collected data precluded a meta-analysis. The flowchart of the literature review is summarized in Figure 1.

Data Synthesis

Owing to substantial heterogeneity in study design, participant populations, motion analysis techniques, outcome measures, and reporting methods, quantitative meta-analysis was not considered appropriate. Instead, a narrative synthesis was performed. The included studies were grouped according to the primary motion analysis modality, including fluoroscopic systems, advanced imaging techniques, motion capture systems, wearable technologies, and functional X-ray analysis. Findings were then qualitatively compared with respect to methodology, clinical application, advantages, limitations, and measurement performance.

Results

Study Selection

A total of 323 studies were identified after removal of duplicates. Initial manual screening of title and abstract was performed. Of these, 258 studies were excluded with the predefined inclusion criteria of studies that assessed and explored or validated novel lumbar spine motion assessment techniques. Overall, 65 reports were sought for retrieval; 5 could not be retrieved, leaving 60 full-text articles for eligibility assessment. Of these, 48 were excluded for the following reasons: lack of evaluation and validation of lumbar motion analysis method (n = 21), insufficient outcome data (n = 15), and irrelevant to lumbar spine motion analysis (n = 12). A total of 12 studies were included after meeting inclusion criteria during full-text review [9,10,12,15-23]. No additional studies were included in our study during citation searching.

Characteristics of Included Studies

Twelve studies met the inclusion criteria and were subsequently grouped by modalities of assessment. A variety of modalities were assessed, and the key findings along with the modality from each of the studies were summarized in Table 1, including dynamic fluoroscopic systems (n = 5), advanced imaging modalities (n = 2), motion capture systems (n = 4), and functional X-ray analysis (n = 1). Most studies were validation or methodological investigations involving healthy volunteers, symptomatic patients, or cadaveric models.

**Table 1:** Summary of literature review of lumbar spine motion analysis VMATM: Vertebral Motion Analysis, FE: flexion-extension, NHS: National Health Service, DM: digitized manual, MRI: magnetic resonance imaging, HASTE: Half-Fourier acquisition single-shot turbo spin-echo, GRE: gradient-echo, DVC: digital volume correlation, LBP: lower back pain, IVD: intervertebral disc, IMU: inertial measurement unit, DOF: degrees of freedom, ICC: intraclass correlation coefficient, FXA: Functional X-ray Analysis, CAD: computer-aided design

Author/year	Study designs	Modality	Sample population	Sample size	Methodology	Key findings
Fluoroscopic systems						
Davis et al. (2015) [9]	Retrospective comparative study	VMA^TM^	Symptomatic spine patients from four neurosurgery facilities and asymptomatic healthy participants	675 individuals (509 symptomatic patients versus 166 asymptomatic participants)	Evaluate VMA versus standard FE radiographs	The VMA^TM ^system showed increased measurement performance in the detection of radiographic instability compared with fluoroscopic FE radiographs.
Harvey et al. (2016) [15]	Experimental observational study	Computer-assisted digitalization and analysis	Former NHS Grampian patients who had undergone lumbar spine fusion or ligament stabilization at least nine months prior	8 participants (7 symptomatic versus 1 asymptomatic)	Clinical measurement and motion analysis using sagittal plane video fluoroscopy	Viable non-invasive technique to assess lumbar spine intervertebral motion. Larger sample size required to validate disease states from motion signatures.
Bifulco et al. (2012) [16]	Methodological study	Continuous description of intervertebral motion	Healthy subjects	Sagittal fluoroscopic image sequences of 3 healthy subjects	Participants lay on their side on a motorized table that performed a passive FE arc (±40°) over ≤24 seconds while video fluoroscopic images were captured at 5 fps	Continuous intervertebral motion analysis may be a different modality to evaluate pathologies and prosthetic implant function. Describes estimation of continuous intervertebral motion with smoothing spline interpolation from functional radiographs.
Wong et al. (2006) [17]	Validation study	Continuous dynamic motion analysis	In vivo: healthy volunteers recruited from the outpatient department of physiotherapy at Queen Elizabeth Hospital, Hong Kong; in vitro: a lumbar spine cadaver model (L4 vertebra)	30 healthy volunteers (16 men and 14 women)	In vitro: evaluated system accuracy on a motor-driven cadaveric L4 vertebra wrapped in soft tissue simulator; in vivo: validated tracking accuracy against expert radiologist markings across 200 frames (5 subjects); in vivo kinematics: measured continuous dynamic FE range of motion using upright video fluoroscopy	Image analysis system used to track in vivo spinal motion of lumbar vertebrae in video fluoroscopy.
Yeager et al. (2014) [18]	Prospective reliability study	VMA^TM^	61 subjects across 7 centers	205 intervertebral levels total (L1-L2 to L5-S1) derived from single lateral motion image sequences (flexion or extension) of those 61 subjects	Automated computer-assisted measurements using the KineGraph VMA system versus a DM technique using medical imaging software	Intervertebral measurements were calculated with a computer-assisted analysis system. Demonstrated reduced variability compared to manual technique of measurement.
Advanced imaging						
Walter et al. (2021) [10]	Retrospective study	Dynamic MRI	Large academic outpatient imaging center between January 2017 and July 2018 for dynamic lumbar or cervical spine MRI with corresponding FE radiographs performed within 2 months	16 lumbar spines	Evaluation of real-time dynamic 3-T MRI sequences (HASTE, continuous radial GRE, and True FISP) compared against a gold standard	Dynamic MRI demonstrated high diagnostic accuracy for functional spinal assessment.
Tavana et al. (2023) [19]	Methodological study	MRI combined with DVC	Healthy human volunteers presenting with no history of LBP	6 healthy subjects: lumbar spine vertebral levels and intervertebral discs from L1 to S1 evaluated per subject	Development and evaluation of a non-invasive tool combining 3T clinical MRI with DVC to measure 3D vertebral translations and IVD strains in vivo	Novel tool that combines DVC and 3T-MRI to assess lumbar kinematics
Motion capture systems						
Moon et al. (2023) [20]	Validation study	IMU	Healthy adult human subject	1 individual	A three-IMU wearable cluster was developed to track 3D lumbopelvic motion, using a four-DOF robotic spine simulator and matrix-based pattern recognition to generate and compare movement templates	Three-IMU cluster system proposed to address limitations of a single IMU-based system
Chang et al. (2022) [21]	Validation study	DorsaVi^TM^ IMU versus Vicon (marker motion capture)	Adults (>18 years of age) employed in manual handling occupations	12 participants (9 with low back pain and 3 without low back pain)	Assessed the agreement between DorsaVi^TM^ version 6 wearable IMU sensors and the 19-camera Vicon motion analysis system (the gold standard reference) during dynamic movement (5 symmetrical and 20 asymmetrical box lifting tasks)	Acceptable concurrent validity between both systems in assessing lumbar motion in multiplane lifting movements.
Yang et al. (2005) [22]	Validation study	Fastrak ® - electromagnetic sensor	Older adult volunteers (72 ± 4 years old)	31 participants (10 men and 21 women)	Compared skin-mounted electromagnetic tracking sensors (Fastrak system) against conventional lateral radiographs (the gold standard reference) to assess measurement accuracy (rotational angles and translational movements) during static trunk positions (neutral upright, full flexion, and full extension)	Rotational angle of vertebrae can be predicted with acceptable accuracy. However, accuracy of translational intervertebral movements was not acceptable.
Hani et al. (2022) [23]	Reliability study	Wearable IMU system	Asymptomatic/healthy adult volunteers aged 18-64 years	20 healthy participants (10 men and 10 women)	Wearable IMU-based lumbar spine motion system (Xsens MTw2) evaluating lumbar kinematics during standardized functional movements in the axial, lateral, and sagittal planes. Intra- and inter-rater reliability were assessed using ICC	Demonstrated moderate-to-excellent intra-rater (ICC: 0.84-0.93) and moderate-to-good inter-rater reliability (ICC: 0.66-0.83), supporting the reliability of IMU-based lumbar motion assessment.
Functional X-rays						
Schulze et al. (2011) [12]	Validation study	Radiograph with FXA^TM^ augmented analysis	CAD model phase: synthetic 2D images derived from a 3D human lumbar spine CAD model (L1-L5); in vitro phase: lumbar functional spinal units (L3-L4) obtained from 3-4-month-old calves (bovine specimens)	CAD phase: 1 synthetic model tested across 6 rotation steps (0.1°-5°); in vitro phase: 6 bovine specimens (N = 6, L3-L4) tested across 11 flexion/extension steps (0.1°-9°)	Theoretical validation: verified algorithms using synthetic 2D images from a 3D human lumbar model (L1-L5) rotated 0.1°-5°; in vitro setup: tested six bovine lumbar units (L3-L4) in a robot arm with soft tissue and facets removed to avoid deformation; intervention and measurement: the robot moved specimens 0.1°-9° in flexion/extension; movement accuracy was evaluated using functional radiographs (FXA^TM)^ and optical motion tracking	FXA^TM^ was validated functional X-ray analysis for assessing lumbar range of motion, center of rotation, and implant migration using both synthetic models and bovine lumbar specimens, demonstrating high measurement accuracy across experimental conditions.

Motion Analysis Modalities

Dynamic fluoroscopic techniques consistently demonstrated improved measurement accuracy and reproducibility compared with conventional flexion-extension radiographs [9,15-18]. MRI-based methods provided radiation-free functional assessment of lumbar kinematics [10,19], whereas wearable IMU systems showed good-to-excellent reliability for objective lumbar motion assessment [20-23]. Functional X-ray analysis and computer-assisted image analysis also demonstrated promising accuracy for measuring lumbar range of motion and intervertebral motion [12]. Overall, these findings indicate a progressive shift toward more objective, dynamic, and clinically applicable methods of lumbar spine motion analysis.

Quality Assessment

Study quality was assessed using the Quality Assessment of Diagnostic Accuracy Studies-2 (QUADAS-2) tool [24], a design appraisal tool. This appraisal tool was selected because the primary objective of the included studies was to evaluate the validity, accuracy, reliability, or measurement performance of lumbar spine motion analysis techniques against established comparator methods rather than to investigate therapeutic interventions. Therefore, QUADAS-2 was considered more appropriate than tools designed for intervention or observational studies, as it specifically evaluates methodological bias related to patient selection, index test conduct, reference standards, and study flow. Detailed scoring results are presented in Table 2.

**Table 2:** Quality appraisal of the included studies using QUADAS-2 QUADAS-2: Quality Assessment of Diagnostic Accuracy Studies-2

Author/year	Patient selection	Index test	Reference standard	Flow and timing	Patient applicability	Index test applicability	Reference standard applicability	Overall risk
Davis et al. (2015) [9]	Low	Low	Low	Low	Low	Low	Low	Low
Harvey et al. (2016) [15]	Low	Low	Unclear	Unclear	Low	Low	Unclear	Unclear
Bifulco et al. (2012) [16]	Low	Low	Low	Low	Low	Low	Low	Low
Wong et al. (2006) [17]	Low	Low	Low	Low	Low	Low	Low	Low
Yeager et al. (2014) [18]	Low	Low	Low	Low	Low	Low	Low	Low
Walter et al. (2021) [10]	Low	Low	Low	Unclear	Low	Low	Low	Unclear
Tavana et al. (2023) [19]	Low	Low	Unclear	Low	Low	Low	Unclear	Unclear
Moon et al. (2023) [20]	Low	Low	Low	Low	Low	Low	Low	Low
Chang et al. (2022) [21]	Low	Low	Low	Low	Low	Low	Low	Low
Yang et al. (2005) [22]	Low	Low	Unclear	Unclear	Low	Low	Unclear	Unclear
Hani et al. (2022) [23]	Low	Low	Unclear	Low	Low	Low	Unclear	Unclear
Schulze et al. (2011) [12]	Low	Low	Low	Low	Low	Low	Low	Low

Discussion

The clinical need for accurate, reproducible measurements of the lumbar spine has spurred new developments in the area as highlighted above. This review highlights the diversity of available methodologies, each with distinct advantages and limitations that influence their clinical applicability.

Static Radiographs: The Foundation of Assessment

The foundation of the assessment of lumbar spinal motion is the functional static radiographs [8]. These static flexion-extension radiographs remain the cornerstone of lumbar motion assessment in clinical practice primarily due to their accessibility, cost-effectiveness, and established clinical familiarity [6,25,26]. However, the inter- and intra-observer variability inherent in manual radiographic measurements represents a fundamental challenge [5,27].

Furthermore, static radiographs provide only endpoint measurements of maximum voluntary flexion and extension, failing to capture the dynamic nature of spinal motion under physiological loads. This limitation is particularly relevant when assessing spinal biomechanics and segmental motion, especially in conditions where dynamic instability or implant performance is of clinical interest [28-30]. However, developments in computer-aided design (CAD) software, such as FXA^TM^ augmented analysis as described by Schulze et al. [12], show promise that computing will be able to augment simple imaging modalities to extract information from existing imaging methods that we were previously not able to analyze.

Dynamic Fluoroscopic Techniques: Bridging Static and Functional Assessment

Dynamic fluoroscopic techniques represent an important advancement over conventional flexion-extension radiographs by enabling continuous assessment of lumbar motion throughout the movement cycle. Studies by Harvey et al. [15], Bifulco et al. [16], Davis et al. [8], and Yeager et al. [18] consistently demonstrated that combining fluoroscopy with computer-assisted image analysis improves measurement accuracy, reproducibility, and detection of radiographic instability while reducing observer variability. These findings suggest that advanced image-processing techniques can enhance the diagnostic capability of existing fluoroscopic imaging systems. However, radiation exposure, two-dimensional image acquisition, and the need for specialized software remain barriers to routine clinical implementation [9,15,16,18].

However, dynamic fluoroscopic techniques present their own challenges. The increased radiation exposure compared to static radiographs raises concerns for routine clinical use, particularly for serial monitoring [31]. Additionally, most fluoroscopic systems provide only two-dimensional analysis, limiting assessment to sagittal and coronal planes while missing important axial rotation and coupled motion patterns. The requirement for specialized software and technical expertise may also limit widespread clinical adoption, especially in low-resource institutions.

Dynamic MRI: Non-invasive Functional Assessment

The emergence of dynamic MRI techniques, as demonstrated by Walter et al. [10], represents an important advancement in non-invasive lumbar spine motion assessment. In the present review, only the findings from the 16 lumbar spine patients included in the study by Walter et al. [10] were considered, while the cervical spine cohort was excluded to maintain consistency with the review objective. Dynamic MRI sequences offer the unique advantage of soft tissue visualization combined with kinematic analysis, potentially providing insights into disc behavior, facet joint motion, and surrounding soft tissue dynamics during movement [32-34].

One novel approach described by Tavana et al. [19], combining digital volume correlation (DVC) with 3T-MRI, represents a cutting-edge development in lumbar kinematic assessment. This technique potentially addresses limitations of other imaging modalities by providing three-dimensional kinematic data without ionizing radiation from X-rays or fluoroscopic systems nor using any invasive marker placement. However, the clinical validation and widespread applicability of these advanced MRI techniques remain to be established through larger studies.

Motion Capture Systems: Laboratory Precision Meets Clinical Reality

The application of marker-based motion capture systems to lumbar spine assessment represents an attempt to bring laboratory-grade precision to clinical evaluation. The comparison by Chang et al. [21] of wearable IMU systems (DorsaVi) with Vicon marker-based systems demonstrates acceptable concurrent validity, suggesting that these technologies may bridge the gap between research capabilities and clinical practicality. These marker-based systems offer the advantage of real-time, three-dimensional motion analysis. The ability to assess functional movements under various loading conditions provides insights into spinal biomechanics that static or limited dynamic techniques cannot capture. However, the inherent limitations of skin marker artifacts, particularly in the lumbar region where soft tissue movement can significantly influence measurements, remain a persistent challenge [35-37]. This was demonstrated in the study by Yang et al. [22], where it was shown that the Fastrak® was not appropriate to predict translational movements of osteoporotic spines, where it was postulated that aged individuals were likely to have fatty bagged back, which resulted in inaccurate differences in the results that were obtained during the study.

Wearable Technology: Real-World Monitoring Potential

Wearable IMU systems represent a promising radiation-free approach for objective lumbar spine motion assessment during functional activities. Across the included studies, Hani et al. [23] reported moderate-to-excellent intra-rater reliability (intraclass correlation coefficient (ICC): 0.84-0.93) and moderate-to-good inter-rater reliability (ICC: 0.66-0.83), while Chang et al. [21] demonstrated acceptable concurrent validity between wearable IMU sensors and the Vicon motion analysis system. Together with the findings of Moon et al. [20], these studies support the potential of wearable IMU systems as reliable tools for objective lumbar spine motion assessment.

Clinical Implications

The diversity of available techniques highlights that no single method currently provides optimal balance of accuracy, practicality, and clinical relevance. The choice of assessment method must be tailored to specific clinical questions and available resources. For routine clinical monitoring, static radiographs may remain appropriate despite their limitations, while research applications or complex cases may benefit from more sophisticated techniques.

The future of lumbar motion analysis likely lies in the integration of multiple modalities, leveraging the strengths of each technique while compensating for individual limitations. The combination of static radiographic screening with targeted dynamic assessment, supported by wearable monitoring for functional validation, may provide comprehensive evaluation frameworks that are both clinically practical and scientifically rigorous.

Future Advancements

Standardization of imaging protocols and measurement of spinal range of motion: The lack of standardized protocols and objective assessment across techniques makes comparison challenging. Future research should focus on establishing standardized measurement protocols, validating techniques against patient-reported outcomes (PROs), and developing integrated assessment frameworks that combine the strengths of multiple modalities. These advancements must be carefully validated against established clinical benchmarks to ensure their reliability and clinical relevance.

Advances in dynamic kinematic analysis: Continued refinement of dynamic imaging, motion capture, and wearable sensor technologies may improve the assessment of lumbar spine biomechanics under functional conditions. Future studies should focus on validating these technologies and determining their clinical utility in the evaluation of degenerative spinal disorders and treatment outcomes.

Correlating biomechanical data with outcomes: Future research should focus on correlating biomechanical findings with patient-reported outcomes, functional recovery, disease progression, and, where applicable, long-term implant performance. This would allow establishment of a threshold to treat. Furthermore, in addition to PROs, other objective outcomes such as progression of adjacent disc disease, implant durability and longevity can be assessed in relation to range of motion.

Expansion of clinical applications: Future studies should investigate the role of lumbar motion analysis across diverse clinical settings, including spinal instability, degenerative lumbar disorders, postoperative assessment following both fusion and motion-preserving procedures, rehabilitation monitoring, and long-term functional evaluation. The integration of advanced motion analysis techniques into routine clinical practice may facilitate more individualized treatment strategies.

Strength

This review systematically evaluated contemporary lumbar spine motion analysis techniques using predefined eligibility criteria and a structured methodological quality assessment. The inclusion of multiple imaging and motion analysis modalities provides a comprehensive overview of current approaches.

Limitations

This systematic review has several limitations. This review was limited to PubMed and English-language publications; therefore, relevant studies indexed in other databases, including those evaluating emerging motion analysis techniques, may not have been identified. The included studies were heterogeneous in design, modality, and outcome reporting, which limited direct comparison across methods and precluded meta-analysis. Many of the studies were small validation or feasibility studies, which reduces certainty and generalizability. In addition, checklist-based quality appraisal provides a structured comparison but may not capture all sources of bias relevant to lumbar motion analysis research.

## Conclusions

Lumbar spine motion analysis remains fundamental to understanding spinal biomechanics and evaluating treatment outcomes. Conventional radiographs continue to be widely used, while dynamic fluoroscopy, MRI, functional X-ray analysis, and wearable IMU systems offer complementary advantages for assessing lumbar kinematics. Continued standardization of imaging protocols and further validation of emerging technologies are needed to facilitate broader clinical application.
